# Religion and mental health: a narrative review with a focus on Muslims in English-speaking countries

**DOI:** 10.1192/bjb.2020.34

**Published:** 2021-06

**Authors:** Ahmed Ibrahim, Rob Whitley

**Affiliations:** 1 Independent Scholar, Montreal, Canada; 2 Department of Psychiatry, McGill University, Canada

**Keywords:** Religion, mental health, Islam, clergy, Muslim mental health

## Abstract

Numerous commentators have noted a historic ambivalence between religion and psychiatry. However, a growing body of evidence indicates an association between mental health and various religious activities, both private and public. As such, there are growing calls for greater religious sensitivity among mental health clinicians, to help unlock the potentially healing aspects of religiosity. So far, most literature from English-speaking countries has focused on Christianity and mental health, with little attention paid to Muslim mental health. This is the fastest growing religion in English-speaking countries, and the mental health of Muslims in these countries is under-researched. As such, the present paper summarises new directions in the mental health and religion literature, with a focus on the mental health of Muslims in English-speaking countries.

Numerous commentators have noted a historic ambivalence between religion and psychiatry.^1–4^ This can be traced back to the writings of formative figures in the early decades of psychiatry; for example, Freud argued that religion was a delusional infantilism that could be disabused through therapy.^5^ Other magisterial figures such as Ellis and Skinner also considered some aspects of religion to be antithetical to positive mental health.^6,7^

More recent studies indicate that this ambivalence continues to the present day. For example, one study found low levels of religiosity among psychiatrists compared with other physicians,^8^ while another study found a lack of integration of religious variables into psychiatric research.^9^ This may signify a continued uneasiness within psychiatry about incorporating aspects of religion into psychiatric research and practice.

## Religion and mental health

That said, a growing body of research suggests a positive association between mental health and religious activities, known in the social sciences as ‘religiosity’.^10^ This literature indicates that religiosity is positively associated with prevention and recovery.^1,11^ For example, a large corpus of research indicates that religiosity is moderately associated with greater well-being, lower rates of depression and anxiety, and lower rates of suicide.^12–14^ Similarly, other studies suggest that both public and private religiosity is associated with recovery from a range of mental illnesses, including depression, schizophrenia and substance use disorder.^15–17^ Of note, many of the studies examining religiosity and mental health are cross-sectional, meaning that reverse causation is possible. However, the consistency of the findings has led researchers to further investigate religiosity to examine specific factors that may be associated with mental health.

## Public and private religiosity

Some scholars divide religious practice into private (interiorised) or public (exteriorised) religiosity.^12,13^ Private religiosity refers to factors occurring alone or in the family home, for example, reading scripture or private prayer. Public religiosity refers to communal religious activities such as attending a place of worship or group study of a sacred text. Some religious activities are transversal across both those domains. For example, adherence to a moral code or a deep religious faith could manifest in both public and private domains.

Research indicates that these aspects of religiosity are associated with better mental health. For example, some anthropological literature indicates that participation in rituals can influence mental health through release of positive emotions.^18^ Moreover, public religiosity provides a community of believers, which can reduce loneliness and bring beneficial social support.^12^ Private religiosity can help solidify family cohesion and provide ontological security during difficult life situations and transitions.^17^ All this can provide a sense of meaning and purpose, as well as a hopeful yet realistic view of life, which can be an ongoing resource for resilience, recovery and positive mental health.^19,20^

## Negative aspects of religiosity

In acknowledging these positive aspects of religiosity, it is important to consider other research indicating the potential harmful aspects of religiosity. For example, some research indicates that religiosity can lead to excessive feelings of guilt, fear, shame and death anxiety.^21,22^ Similarly, high levels of religiosity may lead some people to consult religious leaders in lieu of seeking help from a mental health professional in the presence of mental distress.^23^

Some religious leaders may encourage such consultation, believing themselves to be better placed than clinicians in the process of ‘diagnosing’ distress and facilitating mental healing. For example, some faith leaders may impute mental illness to a spiritual weakness or insufficient religiosity. Likewise, mental illnesses may be attributed by these leaders to supernatural forces such as the ‘evil eye’ or ‘spirit possession’. This can lead to religiously inspired actions such as exorcism and the discouragement of mental health service utilisation.^23,24^ Research on Muslim chaplains in the USA, for instance, found a low rate of referral of congregants to mental health professionals among chaplains.^25–28^ This indicates the need for increased understanding in order to build better bridges between Muslims and mental health services. The next section thus focuses on Islam, Muslims and mental health.

## Islam and mental health

As English-speaking countries become more diverse, with increasing proportions of the population following non-Christian faiths, the relationship between mental health and religion becomes more complex. Most notably, the Muslim population of many English-speaking countries is rising, with Muslims now making up 3.2% of Canada's population,^29^ compared with 5% in the UK.^30^ According to the Pew Research Center, there were about 3.5 million Muslims living in the USA in 2017, making up around 1% of the population. By 2040, Muslims will replace Jews as the second largest religious group in the USA.^31^

There may be a differential effect on mental health according to religious affiliation, belief and practice; however, this has been understudied. For example, religions have many commonalities but also many differences.^32^ These differences manifest themselves in a variety of ways, including notions of morality, theodicy and supernatural intervention.

These differences may have a direct influence on seeking professional mental healthcare, for instance, by channelling people towards religious solutions to the exclusion of seeking psychiatric help. One broad difference between religions relates to the notion of spirit possession.^33^ Possession refers to the belief that an individual has been entered by an alien spirit that controls or alters their actions, manifesting as an altered state of consciousness.^33^ It is often found among Muslims but less so among Jews or Christians in English-speaking countries. Possession is frequently used as an explanatory model in some religious communities and among religious individuals to interpret illnesses such as epilepsy, panic and depression.^33,34^

In Islam, supernatural beings that can take possession of humans are known as jinn, who are conceived as a race of intelligent beings that possess rational faculties. They marry, reproduce and die. According to Islamic doctrine, unlike humans, they have extraordinary powers. They can take different shapes, such as birds, animals and humans, and can move instantly from one place to another.^35^ Jinn are discussed in Islam's scripture, the Qur'an, as well as in the hadith literature.

Although the majority of Muslims believe in the presence of jinn, there is heterogeneity of belief regarding how much they can influence the human world.^35^ Conducting ethnographic interviews with East London Bangladeshis, one study found that the community was split into two main groups. The older generation believed in the power of jinn and often attributed sickness to jinn, whereas the younger second- and third-generation Bangladeshis considered their parents' beliefs to be superstitious and ‘non-Islamic’. Despite the younger generation's sceptical comments about the older members of their community, almost all informants cited instances related to jinn's malevolent power.^35^

The aforementioned study of Bangladeshi Muslims in East London^35,36^ also indicated that some sufferers of ‘jinn’ and their families are likely to seek help from their religious leaders rather than mental health clinicians, even though the latter may be better placed to diagnose and treat any underlying mental health issues. This could be because people commonly perceive religious solutions as the answer to what they perceive to be religious problems.^17^ As such, Littlewood advises psychiatrists to be sensitive to such cultural beliefs by not contradicting any statements made by the patient and their family about jinn or spirit possession.^33^ Instead, he recommends involving ‘culture brokers’ such as an imam from the culture in question to provide contextual information on local beliefs and practices. This could positively affect the therapeutic alliance when authorised by the patient.^15^

It is important to recognise that broad religious traditions such as Islam contain much internal heterogeneity. Anthropologists have tried to capture this heterogeneity by devising the concepts of Great and Little Tradition. The Great Tradition in Islam is the textual, intellectual tradition of the towns, whereas the Little Tradition is the traditional, ritualistic religion of the countryside.^37^ Beliefs in exorcism and jinn tend to be more prevalent within the Little Tradition.^38^ Importantly, both these traditions may exist among immigrant and minority communities in English-speaking countries.

This example of belief in Jinn indicates how religious variables can influence explanatory models and mental health service utilisation. These religious beliefs and explanatory models often traverse time and geography. This brings us to the second part of this paper, which examines practices that can integrate religious variables into clinical care.

## Integrating religion into clinical care

Clinicians can harness helpful aspects of religiosity to foster recovery in patients where appropriate. Moreover, clinicians and clergy can collaborate in the holistic care of patients. However, many clinicians are understandably wary of engaging in such activities, perhaps owing to the aforesaid complexities, as well as unfamiliarity with the diversity of religious experience.^39^

Existing examples of positive collaboration tend to stem from cooperation between clinicians and Christian clergy. This is not necessarily because of anything inherently conducive to this form of cooperation within Christian theology or praxis; it may simply be due to strength of numbers in English-speaking countries. One example of clergy–clinician collaboration is Hope Haven, a private agency in Iowa that has been providing psychosocial rehabilitation services for many years.^11^ The agency combines spirituality with mental health services in various ways. For example, the Religious Services department at Hope Haven seeks to engage area churches in welcoming and including people with psychiatric disabilities into the life of the church.^40^ The agency offers daily devotions for patients, as well as spiritual support and prayer for those going through difficult situations. In a similar vein, Muslims in the USA and UK have established small-scale centres of psychotherapy such as the Khalil Centre and Ihsan Centre, where Islamic spirituality is integrated into care.^41^ However, such initiatives remain isolated examples.

This raises the question of what can be done to better equip everyday clinicians in routine practice to deal with religious patients and religious issues, especially those from minority faiths such as Islam. Some researchers have speculated whether mental health practitioners should receive training in the spiritual and religious beliefs of major religions so that they can better distinguish religious beliefs from psychopathology.^42,43^ This may be impractical, given the heterogeneity and diversity within and between religions previously described.

An alternative approach is the adoption of a set of attitudes and processes that facilitate the integration of spirituality and religion into clinical care. This could involve working with ‘culture brokers’ such as chaplains or community-based key informants in the treatment of religious patients, depending on the spiritual profile and wishes of the patient in question. This is the approach taken by the Cultural Consultation Service in Montreal, which has a bank of culture brokers that can be accessed by clinicians facing complex religious issues in the treatment of patients.^44^ These culture brokers can offer perspectives and interpretations based on their locally grounded community experience, which may be especially effective if the culture broker is recommended by the patient and involved in their follow-up care. This approach can be a useful adjunct to standard clinical care; however, the provision and training of culture brokers can be a difficult task, meaning that more practical approaches are often necessary.

Instead of relying on abstract nomothetic knowledge or the intervention of third parties, a more practical approach may involve clinicians making conscious efforts to gain an idiographic understanding of the patient's religious worldview during the clinical consultation. Importantly, researchers have developed a number of simple and generic tools and procedures that clinicians can use to elicit information about patient religiosity (or lack thereof), sometimes known as a ‘spiritual assessment’. These tools can be used by psychiatrists in clinical settings to enhance understandings and decision-making, and can be applied to Muslims as well as others.

This includes the Outline for Cultural Formulation and the Cultural Formulation Interview (CFI) contained in the DSM-5.^45^ Supplementary modules to the core CFI include the ‘Spirituality, Religion, and Moral Traditions’ module, which provides 16 useful questions for the deep and meaningful probing of religious issues where appropriate.^46^

Another of these well-known tools is known as the Faith, Importance, Community, and Address (FICA) instrument.^47^ The FICA inquires into the following four domains: (a) *Faith and belief*, ‘Do you have spiritual beliefs that help you cope with stress?’; (b) *Importance*, ‘What role do your beliefs have in regaining health?’; (c) *Community*, ‘Are you part of a religious or spiritual community? If so, is this of support to you and how?’; and (d) *Address* in care, ‘How would you like me as your healthcare provider to address these issues in care?’.

Importantly, the short and neutrally posed questions allow atheists and non-religious people to quickly express a lack of interest in these issues and move onto other topics. Of note, there has been little research on the use and effectiveness of such tools and instruments among Muslim patients. This is an important area for future research.

Interestingly, some researchers have proposed specific procedures for clinicians working with Muslim patients. Abu Raiya and Pargament^48^ proposed a series of recommendations including: (a) asking about the place of religion in patients’ lives; (b) educating themselves about basic Islamic beliefs and practices; (c) helping patients draw on Islamic religious coping methods; (d) referring to a clergy member if appropriate; and (e) participating in educating Muslims about mental health. These recommendations overlap with the above-described generic advice for clinicians dealing with religious patients and could be a useful tool for working with Muslim patients.

All these tools have the potential to foster what has been termed ‘existential recovery’, defined as ‘having a sense of hope, empowerment, agency, and spiritual well-being’.^49^ For example, clinicians may refer an isolated patient of faith to a sympathetic chaplain for spiritual and social support, who may in turn link the patient to a community of believers. However, such actions must be tailored to individual need and preferences, and may be more difficult for patients who are members of minority faiths such as Islam, where access to Muslim chaplains is more limited.

Indeed, research shows that Muslim chaplains are underutilised in English-speaking countries such as the USA.^25–27^ In Britain, one study of the provision of spiritual and pastoral care facilities in a high-security hospital revealed that demand for pastoral care could be significantly higher among Muslim patients compared to Christian patients. With one Muslim chaplain employed part-time, the authors argued that the allocation of chaplaincy resources should be re-examined in light of the multi-faith nature of modern Britain.^50^ This is an area in need of further discussion and research.

In addition, clergy–clinician collaboration could be better attained by giving mental health training to clergy and other religious leaders. In fact, there are now a number of pastoral counselling programmes training clergy and others to help people with mental health issues, using evidence-based psychotherapies nested within a religious framework. Such programmes can be found at prestigious US universities including New York University and Northwestern University. Again, such initiatives have predominantly involved Christian clergy; training of Muslim religious leaders is lacking.^27^ Likewise, counselling courses are offered to the clergy by the Association of Christian Counsellors in the UK, among others, but not for Muslim clergy *per se*.

Despite these efforts, there is a need for further research and action in this regard. For example, a study of British clergy representing Christianity, Judaism and Islam revealed that most members of the clergy had received little or no training in mental health as part of their ministry training.^4^ The clergy members interviewed seldom differentiated between psychotic illness and common mental disorders such as depression and anxiety. Some members of the clergy interpreted unusual or disturbing behaviour as a religious problem provoked by a curse, witchcraft or spirit possession. In these cases, prayers and exorcism were considered an appropriate response.^51^

It is unlikely that mental health training for the clergy will cause these religious interpretations to disappear, because people may draw upon more than one explanatory model at a time to explain distress. However, training may give a more holistic understanding, and research indicates that people often hold coexisting religious and psychiatric explanatory models of mental illness, which can lead them to use various modalities of healing in cases of mental distress.^17,52^

Indeed, it is important to communicate to clinicians and clergy that religious and psychiatric intervention is not an ‘either-or’ scenario; both deployed simultaneously could produce effective results. For example, anthropological research indicates that certain rituals such as prayer may be beneficial to the healing and recovery of some individuals.^18^ Thus, it is not advisable to reject such practices out of hand without learning about the preferences and worldviews of individual patients. This is where ‘spiritual assessment’ tools can be useful, even necessary.

## Conclusion

There is growing evidence that the influence of religion on mental health is largely positive. This research supersedes outdated notions perpetuated by figures such as Freud about the negative effects of religion on mental health. Moreover, this growing evidence gives impetus to new models of cooperation between religious leaders and mental health professionals.

In an ideal world, this would involve a bidirectional system of cooperation and education. On the one hand, clergy could receive basic training and education in mental health. This could improve understanding of mental illness and increase referrals from clergy to mental health professionals. Similarly, educational and public outreach campaigns could be targeted at religious and minority communities, with cooperation and participation from the communities themselves. On the other hand, there is still a need for better education of mental health professionals in religious matters. This includes training in areas such as taking a spiritual history and working with culture brokers and community religious leaders. Such training could be co-delivered by clinical experts and religious leaders.

At the policy level, administrators may reconsider their chaplaincy resource allocation to ensure that minority faiths, whose adherents tend to have greater religious counselling needs, are proportionately resourced. Importantly, new action and research that responds to the growing religious diversity of English-speaking societies is necessary, with particular focus on the growing and heterogeneous Muslim community to ensure that policy and practice are based on evidence rather than stereotypes. This could ultimately lead to more supportive and tailored treatment options that harness, rather than ignore, patient religiosity and spirituality, thereby promoting a holistic recovery in religious patients.

## About the authors

**Ahmed Ibrahim** is an independent scholar in Montreal, Quebec, Canada. **Rob Whitley** is an Associate Professor in the Department of Psychiatry, McGill University, and a Research Scientist at the Douglas Hospital Research Centre, Montreal, Quebec, Canada.
